# CCR2- and Flt3-Dependent Inflammatory Conventional Type 2 Dendritic Cells Are Necessary for the Induction of Adaptive Immunity by the Human Vaccine Adjuvant System AS01

**DOI:** 10.3389/fimmu.2020.606805

**Published:** 2021-01-14

**Authors:** Cedric Bosteels, Kaat Fierens, Sofie De Prijck, Justine Van Moorleghem, Manon Vanheerswynghels, Caroline De Wolf, Aurélie Chalon, Catherine Collignon, Hamida Hammad, Arnaud M. Didierlaurent, Bart N. Lambrecht

**Affiliations:** ^1^Laboratory of Immunoregulation and Mucosal Immunology, VIB-UGent Center for Inflammation Research, Ghent, Belgium; ^2^Department of Internal Medicine and Pediatrics, Ghent University, Ghent, Belgium; ^3^GSK vaccines, GSK, Rixensart, Belgium; ^4^Department of Pulmonary Medicine, Erasmus University Medical Center Rotterdam, Rotterdam, Netherlands

**Keywords:** AS01, vaccine, adjuvant, dendritic cell, inf-cDC2, CD64, MAR-1, Fc receptor

## Abstract

The Adjuvant System AS01 contains monophosphoryl lipid A (MPL) and the saponin QS-21 in a liposomal formulation. AS01 is included in recently developed vaccines against malaria and varicella zoster virus. Like for many other adjuvants, induction of adaptive immunity by AS01 is highly dependent on the ability to recruit and activate dendritic cells (DCs) that migrate to the draining lymph node for T and B cell stimulation. The objective of this study was to more precisely address the contribution of the different conventional (cDC) and monocyte-derived DC (MC) subsets in the orchestration of the adaptive immune response after immunization with AS01 adjuvanted vaccine. The combination of MPL and QS-21 in AS01 induced strong recruitment of CD26^+^XCR1^+^ cDC1s, CD26^+^CD172^+^ cDC2s and a recently defined CCR2-dependent CD64-expressing inflammatory cDC2 (inf-cDC2) subset to the draining lymph node compared to antigen alone, while CD26^-^CD64^+^CD88^+^ MCs were barely detectable. At 24 h post-vaccination, cDC2s and inf-cDC2s were superior amongst the different subsets in priming antigen-specific CD4^+^ T cells, while simultaneously presenting antigen to CD8^+^ T cells. Diphtheria toxin (DT) mediated depletion of all DCs prior to vaccination completely abolished adaptive immune responses, while depletion 24 h after vaccination mainly affected CD8^+^ T cell responses. Vaccinated mice lacking Flt3 or the chemokine receptor CCR2 showed a marked deficit in inf-cDC2 recruitment and failed to raise proper antibody and T cell responses. Thus, the adjuvant activity of AS01 is associated with the potent activation of subsets of cDC2s, including the newly described inf-cDC2s.

## Introduction

Recombinant proteins are often combined with adjuvants in vaccines to enhance immunogenicity with the aim of conferring protection against disease. Similar to natural infections, adjuvants trigger the innate immune system, which in turn shapes the nature of the adaptive immune response. The Adjuvant System AS01 is a liposome-based adjuvant that contains two immunostimulants (1). The first compound 3-O-desacyl-4’-monophosphoryl lipid A (MPL) is a detoxified lipid from Salmonella Minnesota LPS and a Toll-like receptor 4 (TLR4) agonist (2). MPL promotes the production of pro-inflammatory cytokines and directly activates antigen-presenting cells (APCs) (3, 4). The second compound, QS-21, is a saponin molecule extracted from the bark of the Quillaja saponaria tree (1). QS-21 enhances antibody responses in humans (5), and cytotoxic CD8^+^ T lymphocyte (CTL) responses in mice through modified antigen cross-presentation by cDCs (6–8). QS‐21 activates innate immunity through destabilization of the lysosomal membrane upon cholesterol‐dependent endocytosis, leading to the activation of the caspase‐1 and Syk pathways (6, 7). By activating multiple innate pathways, both immunostimulants in AS01 synergistically enhance antigen-specific T cell responses producing interferon-gamma (IFN-γ) and antibody switching toward IgG2c antibodies in mice (3, 9, 10). We have previously established that the ability of AS01 to induce a strong T cell response relies on a synergy between MPL and QS-21 (9, 11). The liposomal formulation of AS01 allows to improve the delivery of the immunostimulants and to abrogate the hemolytic activity of QS-21 (12). AS01 is included in the recombinant herpes zoster (Shingrix) and the malaria vaccines (13–15) as well as in other candidate vaccines against viruses and intracellular pathogens, such as HIV and tuberculosis (16, 17).

The key cells to integrate and translate the innate cues elicited by AS01 into antigen-specific effector responses are antigen-presenting cells (APCs), and in particular conventional dendritic cells (cDCs). Dendritic cells are classically subdivided in cDCs, plasmacytoid DCs (pDCs) and monocyte-derived DCs (moDCs or MCs). Based on differences in transcriptional control, function, ontogeny and cell surface markers, mature cDCs are further subdivided into cDC1s and cDC2s (18–20). Functionally, cDC1s have the capacity to present and cross-present antigen to CD8^+^ T cells, whereas cDC2s typically drive naive CD4^+^ T cells toward distinct effector subsets (21). While the cDC1 subset is precisely delineated by the expression of specific markers (such as XCR1), cDC2s seem more heterogenous on a transcriptional level and express surface markers (such as CD172a, CD11b) that often overlap with macrophages and MCs (22). Upon induction of inflammation, tissue-infiltrating monocytes rapidly upregulate major histocompatibility complex class II (MHCII) and CD11c, to become so called MCs (or monocyte-derived DCs), that can be easily confused with cDC2s (23–25). Importantly, we have recently identified an activated cDC2 population, called inflammatory-cDC2 (inf-cDC2) that acquires transcriptional, functional and phenotypical characteristics traditionally defining cDC1s (e.g. CD26, IL-12, and IRF8) and MCs (e.g. surface expression of activating FcγR such as CD64 and MAR-1) in a cell-intrinsic, type I interferon dependent manner. In our work on various antigenic triggers (viruses, allergens, bacteria, helminths) in the lung and skin, we described how these pre-cDC-derived hybrid inf-cDC2s efficiently migrated to the dLN in a CCR7-dependent manner to prime both naive CD4^+^ and CD8^+^ T cells, whereas MCs were unable to do so. Consistent with the expression of activating Fc receptors (i.e. CD64 and MAR-1), inf-cDC2s competently endocytosed antibody–complexed antigen for enhanced Th1 priming (23). Although dependency on the CCR2 chemokine receptor for monocyte bone marrow egress was initially considered as a defining characteristic of monocyte-derived cells (26), we found that inf-cDC2s were also dependent on CCR2, and cautioned that CCR2-dependency did not equalize with monocyte origin (23).

The objective of this study was to assess the role played by the different DC subsets in the orchestration of the adaptive immune response raised against two antigens combined with AS01. We have found that combining immunostimulants in AS01 adjuvant results in recruitment of monocytes and DC subsets cDC1s, cDC2s and inf-cDC2s to the dLN, while DC-like MCs were barely detectable. Finally, our findings indicate that the recently described inf-cDC2 subset, next to cDC1s and cDC2s, is essential to confer both antigen-specific cellular and humoral immunity induced by AS01-containing vaccine.

## Material and Methods

### Mice

The following female mice were used in this study; C57BL/6 mice (aged 6–10 weeks) were purchased from Janvier (France). CD45.1 (Jackson laboratories, Stock No: 002014), Itgax-DTR, OVA-specific CD4^+^ TCR Tg (OTII; Jackson laboratories, Stock No: 004194), OVA-specific CD8^+^ TCR Tg (OTI; Jackson laboratories, Stock No: 003831), *Ccr2^-/-^* (Jackson laboratories, Stock No: 027619) and *Flt3^-/-^* mice were bred in house in specific pathogen-free conditions at the animal facility of Ghent University. Animal husbandry and experiments were ethically reviewed and carried out in accordance with European Directive 2010/63/EU. All experiments were approved by the independent animal ethical committee “Ethische Commissie Dierproeven – faculteit Geneeskunde en Gezondheids-wetenschappen Universiteit Gent”.

### Vaccine Formulations and Mouse Immunization

VZV gE antigen, AS01, MPL, and QS-21 formulations were provided by GSK, Rixensart, Belgium. Ovalbumin (OVA) was from Calbiochem and confirmed to be endotoxin depleted. The gE antigen and AS01 are components of the licensed recombinant Zoster vaccine (Shingrix) and were produced in the same GMP conditions for this study^1^. AS01 is composed of QS-21 (Quillaja saponaria Molina, fraction 21, licensed by GSK from Antigenics LLC, a wholly owned subsidiary of Agenus Inc., a Delaware, USA corporation) and MPL in a liposome formulation made of Di-Oleoyl Phosphatidyl Choline (DOPC) and cholesterol. Mice were immunized at day 0 and, in some experiments received a second dose at day 14. Each vaccine dose was administered as two simultaneous i.m. injections of each 20–25 µl in both left and right gastrocnemius muscles. Depending on the experiment (cfr. figure legends), adjuvant dose per injection site was 1 or 2.5 µg AS01 or 1 µg MPL or 1 µg QS-21, all formulated in the same liposome as used in AS01, and combined with antigen(s) either VZV gE (2 or 2.5 µg per injection site) or OVA (5 µg per injection site) or both VZV gE and OVA together (respectively 2.5 µg and 0.5 µg per injection site).

### *Ex vivo* DC-OTII CD4^+^ and OTI CD8^+^ T Cell Co-Culture

Naive OVA-specific CD8^+^ and CD4^+^ T cells were isolated from spleens of OTI and OTII transgenic mice, respectively, enriched through negative selection for CD8^+^ and CD4^+^ T cells (EasySep) and labeled with Cell proliferation dye eFluor450 (CTV, Thermo Fisher Scientific) according to manufacturer’s instructions. DCs isolated from the dLNs 24 h post-immunization with OVA/AS01 were enriched through negative selection (Dynabeads, Thermo Fishser Scientific). A total of 25x10^3^ OVA-specific T cells were then separately co-cultured for 3 days (OTI) or 4 days (OTII) at various ratios with the distinct migratory cDC subsets purified by cell sorting using a FACSAria (> 95% purity) in sterile tissue culture medium [TCM; RPMI (Gibco) containing 5% fetal calf serum (Bodinco), 1.1 mg/ml β-mercaptoethanol (Sigma-Aldrich), 2 mM L-alanyl-L-glutamine dipeptide (Thermo Fisher Scientific), and 56 μg/ml Gentamicin (Thermo Fisher Scientific)].

### Tissue Sampling and Processing

Mice were euthanized at time points indicated by intraperitoneal (i.p.) injection of sodium pentobarbital. Iliacal LNs were digested in 1.2 ml RPMI (Gibco) containing 20 μg/ml Liberase and 10 U/ml Dnase (Roche) for 20 min at 37°C before passing through a 70 μm cell strainer. Splenocytes were obtained by passing through a 70 µm filter and red blood cells were lysed using ammonium chloride lysis buffer (10 mM KHCO3, 155 mM NH4Cl, 0.1 mM EDTA in MilliQ water).

### Flow Cytometry and Cell Sorting

Single cell suspensions were incubated with a mix of fluorescently labeled monoclonal antibodies (Ab) and/or tetramers (cfr. infra) for 30–45 min at 4°C. To reduce non-specific binding, 2.4G2 Fc receptor Ab (Bioceros NV) was added. Dead cells were removed from analysis, using fixable viability dye eFluor506 or eFluor780 (eBioscience). For intracellular staining cells were fixed and permeabilized using Cytofix-Cytoperm and Perm/Wash (BD Biosciences) according to the manufacturer’s protocol. In order to monitor individual cell divisions of T cells, cells were stained with Cell proliferation dye eFl450 (CTV, Thermo Fisher Scientific) according to the manufacturer’s protocol. Cells were stained with anti-mouse antibodies (see **Supplementary Table**). In a second staining step, biotin-conjugated antibodies were bound by SAV-CF594 (BD Biosciences). Before acquisition, photomultiplier tube voltages were adjusted to minimize fluorescence spillover. Single-stain controls were prepared with UltraComp eBeads (Thermo Fisher Scientific) following the manufacturer’s instructions and were used to calculate a compensation matrix. Sample acquisition was performed on an LSR Fortessa or FACSymphony cytometer equipped with FACSDiva software (BD biosciences). Final analysis and graphical output were performed using FlowJo software (Tree Star, Inc.) and GraphPad Prism 6 (GraphPad Software, Inc.). For sorting of DC subsets and T cells, cells were stained as described and cell sorting was performed on a FACSAria II and III (BD biosciences).

### *Itgax*-DTR Transgenic and WT : *Ccr2^-/-^* Bone Marrow Chimeras

Bone marrow cells were prepared by crushing femurs and tibias in phosphate buffer saline (PBS) followed by filtering through a sterile 70 μm cell strainer. C57BL/6 mice (8–10 wk old) were sublethally irradiated (8 Gy) and, 4 h later, received 2x10^6^ bone marrow cells i.v. from transgenic *Itgax*-DTR mouse donors (27). At least 10 weeks after bone marrow reconstitution and either 12 h before or 24 h after immunization, chimeric mice were depleted of CD11c^+^ cells by an injection of 200 ng DT or PBS (control) in both gastrocnemius muscles. In some experiments the immunization was performed twice with 14 days apart. Competitive CD45.1(WT): CD45.2 *Ccr2^-/-^* and CD45.1(WT): CD45.2 *Ccr2^+/+^* bone marrow chimeras were similarly generated by sublethally irradiating (8 Gy) CD45.1.2 C57BL/6 mice (8–10 wk old), that received 2x10^6^ bone marrow cells i.v. in a 50:50 ratio of CD45.1/CD45.2 donors 4 h later.

### Antigen-Specific Antibody and T Cell Response

Anti-VZV gE IgG concentrations in the serum were measured by ELISA, following a protocol described previously (11, 27). The frequency of SIINFEKL (OVA_257–264_)-specific CD8^+^ T cells was determined in peripheral blood or splenocytes by staining with PE-conjugated H-2Kb-OVA_257–264_ tetramers (Beckman Coulter). Single cell levels of cytokine production by antigen-specific T cell response were measured as previously described (27, 28). Briefly, splenocytes from vaccinated mice (2 x 10^6^ cells/well in 96-well microplate) were stimulated *in vitro* with VZV gE peptides (1 µg/ml; encompassing whole antigen, 15-mers with 11 amino-residue overlap; Eurogentec) or OVA peptides (1 µg/ml; pool consists of 17 peptides selected for H2-Kb epitope content, all are 15-mers with 11 amino-residue overlap; Neosystem) in complete RPMI supplemented with 5% fetal calf serum (FCS, Bodinco). Anti-CD49d (9C10) and anti-CD28 (37.51) antibodies (1 µg/ml each, BD Biosciences) were added to the culture. Antigens-specific stimulation of T cells was monitored by intracellular cytokine staining after 18 h incubation at 37°C in which Brefeldin A (1 μg/ml, BD Biosciences) was added for the last 16 h.

### Cytokine Detection in Serum

Serum was collected 24 h after immunization at stored at -70°C until further analysis. Cytokine protein levels were determined by cytokine-specific beads using the Luminex platform (Millipore).

### Statistical Analyses

Results are expressed as mean ± SEM as indicated in figure legends. Statistical tests were selected based on appropriate assumptions with respect to data distribution and variance characteristics. One- way ANOVA with Sidak correction for multiple testing was used for the statistical analysis of differences between more than two groups. Two-way ANOVA with Sidak post-test was used for the statistical analysis of differences between more than two groups and with 2 different independent variables. Statistical significance was defined as p <0.05. Sample sizes were chosen according to standard guidelines. Number of animals is indicated as “n”. Of note, sizes of the tested animal groups were also dictated by availability of the transgenic strains. Statistical details of experiments can be found in the figure legends. The investigator was not blinded to the mouse group allocation.

## Results

### cDC2 is the Main DC Subset Recruited Upon Administration of AS01 or Its Components

**Figure 1:**
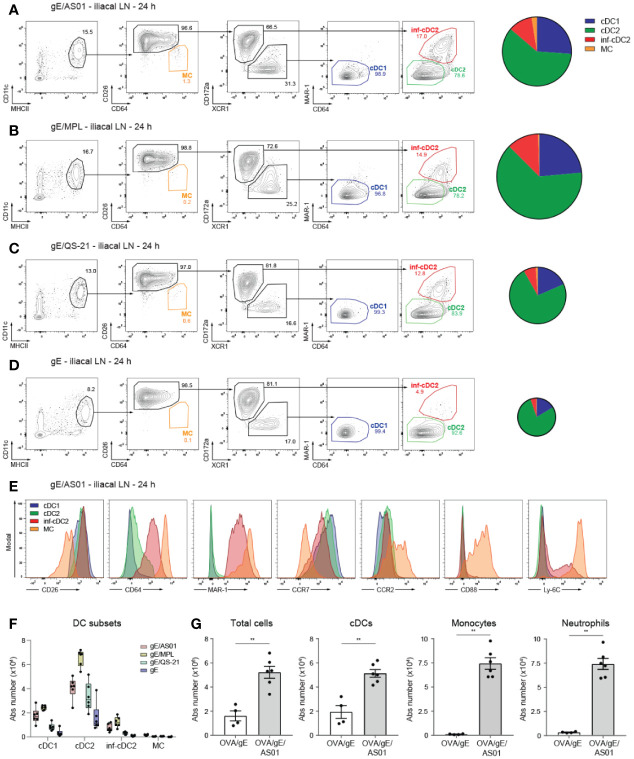
cDC subsets are recruited to the draining lymph node after i.m. AS01 immunization. **(A–D)** Gating strategy of migratory MHCII^hi^ DC subsets pre-gated on live CD3^-^CD19^-^ cells (left panel) and pie charts depicting relative distribution of DC subset (right panel) in the dLN 24 h after i.m. (M. gastrocnemius) immunization with 2 µg VZV gE antigen formulated in 1 µg AS01 **(A)**, MPL **(B)**, QS-21 **(C)**, or buffer **(D)** per injection site. **(E)** Histograms showing surface expression of cDC, monocyte and macrophage markers on different DC subsets in the dLN 24 h after i.m. immunization with gE/AS01 as in **Figure 1A**. **(F)** Absolute number of DC subsets in the dLN 24 h after i.m. immunization with the different compounds as in **Figures 1A–D**. **(G)** Absolute number of total cells, cDCs, monocytes and neutrophils in the dLN 24 h after i.m. immunization with OVA/gE (0.5/2.5 µg) formulated in 2.5 µg AS01 or buffer per injection site. AS01, MPL, and QS-21 are all in liposome. Data are representative of at least 2 independent experiments (n = 4–6 mice per group). Size of the pie chart is proportional to the absolute number of DCs in the dLN **(A–D)**. Error bars indicate mean ± SEM **(G)**. Data analyzed with a Mann-Whitney test **p < 0.01.

We have previously reported in detail the kinetics of the innate immune response in mice after intramuscular (i.m.) injection of AS01-adjuvanted vaccines (27). Neutrophils, monocytes and DCs rapidly [i.e. within 24 h post-injection (pi)] increase in the draining iliacal LN (dLN) after i.m. immunization. Although adaptive immunity induced by AS01-adjuvanted vaccine required CD11c^+^ cells, the relative contribution of the different DC subsets to the T cell priming effect of AS01 or its components was not assessed. Here, we immunized mice by i.m. injection with 2 µg VZV gE formulated in AS01 (gE/AS01), in MPL alone (gE/MPL), or in QS-21 alone (gE/QS-21). Twenty-four hours after immunization, we analyzed the different DCs subsets in the dLN. We employed a universal gating strategy to separate both *Flt3*-dependent cDC1s and cDC2s; and *Flt3*-independent MCs within MHCII^hi^CD11c^+^ cells based on specific surface markers (22). We stained for the dipeptidyl peptidase CD26, identifying all cDCs, whereas MCs were identified as CD26^-^CD64^+^ cells also expressing the complement receptor CD88 (22, 23, 29). XCR1 and CD172a (Sirpα) were used as conserved markers to separate cDC1s from (CD11b^+^) cDC2s, respectively (22). cDC1s characteristically expressed high levels of CD24, while CD11b expression was restricted to cDC2s, inf-DC2s and MCs (data not shown). Additionally, we stained for other activating FcγRs besides CD64, and used MAR-1 staining to mark inflammatory cDC2s and MCs in immunized mice (23, 25, 30, 31) (**Figures 1A–E**).

In mice receiving AS01-adjuvanted vaccine (**Figure 1A**), there was a marked increase in the total number of MHCII^hi^CD11c^+^ migratory DCs in the draining iliac LN 24 h post injection, compared with mice receiving antigen alone (**Figures 1D, F**). This increase was also seen when MPL (gE/MPL) and QS-21 (gE/QS-21) adjuvant components were used (**Figures 1B, C**, **F**). In all conditions of vaccination, the bulk of MHCII^hi^CD11c^+^ cells were composed of CD26^+^ cDCs with only a very limited proportion of CD26^lo^CD64^hi^CD88^hi^ MCs (**Figures 1A–D**, **F**). Within the cDC2 population, MAR-1^+^CD64^+^ inf-cDC2s were only detected in the presence of AS01 or its components. In all groups migratory cDCs were mainly composed of cDC2s, followed by cDC1s and inf-cDC2s (**Figures 1A–D**, **F**).

In conditions of inflammation, myeloid markers often overlap between various cell types. In line with our previous work (23), CD88 was however exclusively expressed by MCs, while Ly6C expression was shared between inf-cDC2s and MCs (**Figure 1E**). Upon activation, antigen-bearing cDCs migrate to the draining LN in a CCR7 dependent manner (32, 33). Surface expression of CCR7 was induced upon immunization with gE/AS01 on cDC1s, cDC2s and inf-cDC2s but not MCs (**Figure 1E**). CCR2 was mainly expressed by MCs, less by cDC2s, while surface CCR2 expression was hardly detectable on migratory cDC1s. Next to DCs, other innate cells such as CD11b^+^Ly6C^+^MHCII^-^ monocytes and CD11b^+^Ly6G^+^ neutrophils were significantly increased in the dLN 24 h pi with AS01-adjuvanted vaccine (**Figure 1G**).

Interestingly, MPL seems to induce DC recruitment more efficiently than AS01 or QS-21, irrespective of the DC subsets, while in contrast, QS-21 performed relatively poorly as compared to AS01 or MPL, especially for cDC1 recruitment (**Figure 1F**). However, the combination of both MPL and QS-21 results in the efficient recruitment of a variety of DC subsets. In contrast to cDCs and although Ly6C^hi^ monocytes heavily infiltrate the dLN upon antigen immunization formulated in AS01 (27), we show here that CD11c^+^MHCII^+^ MCs were barely detectable and expressed low levels of CCR7 in accordance with their poor migratory capacity in other tissues and immunization regimens (23).

### All cDC Subsets can Efficiently Present Antigens After Immunization with AS01-Adjuvanted Vaccines

**Figure 2:**
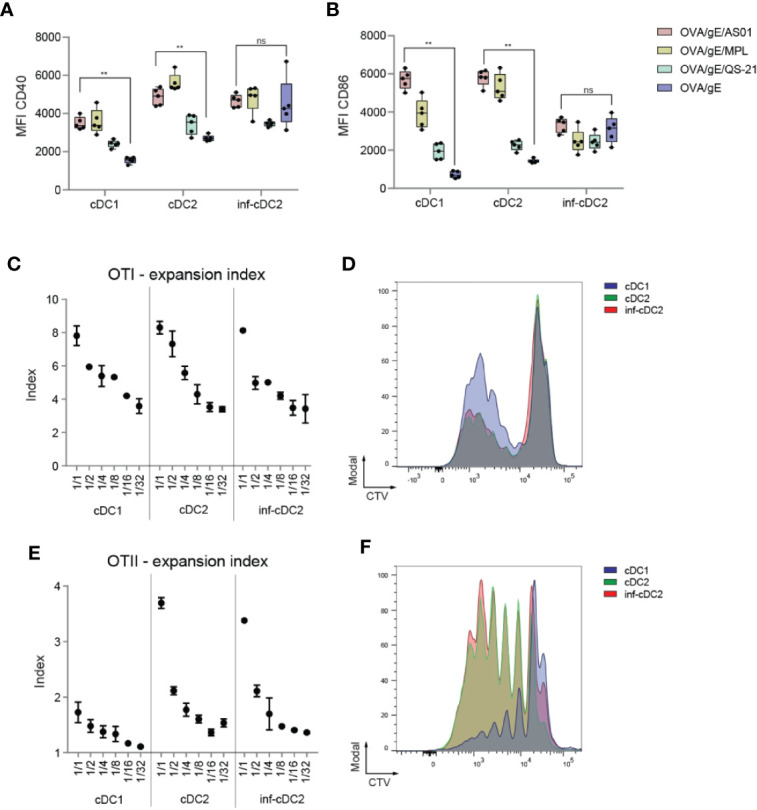
AS01 activated cDCs effectively prime antigen-specific T cells. **(A, B)** Expression of CD40 **(A)** and CD86 **(B)** shown as MFIs by cDC subsets in the dLN 24 h after i.m. (M. gastrocnemius) immunization with OVA/gE antigen (0.5/2.5 µg) formulated in 2.5 µg AS01, MPL, QS-21, or buffer per injection site. AS01, MPL, and QS-21 are all in liposome. Data analyzed with Two-way ANOVA and Tukey’s multiple comparisons test. Data are representative of at least two independent experiments (n = 5 mice per group). **p < 0.01, ns, non-significant. **(C)** Expansion index of CD8^+^ OVA-specific TCR transgenic T cells (OTI) cocultured for 3 days with the different migratory cDC subsets sorted from pooled dLNs (n = 80 mice) 24 h after i.m. immunization with OVA/AS01 (5/1 µg) per injection site. **(D)** Proliferation prolife of CTV-labeled OTI T cells cocultured in 1:4 DC:T cell ratio with the different migratory cDC subsets for 3 days sorted from pooled dLNs (n = 80 mice) 24 h after i.m. immunization with OVA/AS01 (5/1 µg) per injection site. **(E)** Expansion index of CD4^+^ OVA-specific TCR transgenic T cells (OTII) cocultured for 4 days with the different migratory cDC subsets sorted from pooled dLNs (n = 80 mice) 24 h after i.m. immunization with OVA/AS01. (5/1 µg) per injection site **(F)** Proliferation prolife of CTV-labeled OTII T cells cocultured in 1:1 DC:T cell ratio with the different migratory cDC subsets for 4 days sorted from pooled dLNs (n = 80 mice) 24 h after i.m. immunization with OVA/AS01 (5/1 µg) per injection site.

We have shown that association of AS01 to antigen activates the innate immune system and leads to enhanced antigen-specific adaptive immune responses (27). We extended this finding by looking at the capacity of the different cDC subsets to prime naive T cells. As a first step, we investigated the altered activation state of the different cDC subsets. All cDC subsets expressed higher levels of costimulatory molecules such as CD40 and CD86 at 24 h pi with Ovalbumin (OVA)/gE/AS01 compared to antigen alone, except for inf-cDC2s (**Figures 2A, B**). The extent of upregulation of costimulatory molecules by MPL was similar to AS01, and higher compared to QS-21, suggesting that MPL is the main driver of DC activation in AS01. Twenty-four hours after OVA/gE/AS01 immunization, CD40 expression was higher on cDC2s and inf-cDC2s compared to cDC1s (**Figure 2A**), while CD86 was higher on cDC1s and cDC2s as compared to inf-cDC2s (**Figure 2B**). Finally, to assess the individual contribution of the different subsets in Ag presentation, we sorted the subsets from the dLN 24 h pi with OVA/AS01 and measured their ability to present endogenously processed OVA protein to cognate naive CD4^+^ and CD8^+^ T cells *ex vivo*. After 3 days of co-culture at different DC:T cell ratios, cDC1s, cDC2s, and inf-cDC2s all induced the proliferation of OTI cells (**Figures 2C, D**, **Figure 2D** showing CTV profiles at 1:4 DC:T cell ratio). Conversely, cDC2s and inf-cDC2s were more efficient in inducing OTII cell proliferation compared to cDC1s after 4 days of culture (**Figures 2E, F**, **Figure 2F** showing CTV profiles at 1:1 DC:T cell ratio). Thus, all subsets of cDCs, including inf-cDC2s become proficient at presenting AS01-adjuvanted model antigens.

### Depletion of CD11c^+^ Cells *In Vivo* Abrogates Cellular Responses to AS01-Adjuvanted Vaccines

**Figure 3:**
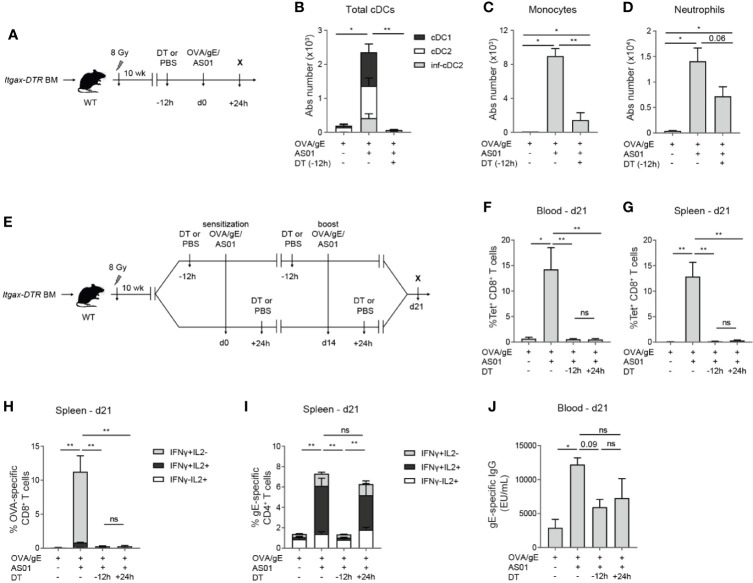
Cellular and humoral adaptive immune responses depend on CD11c^+^ cells. **(A)** Schematic representation of Itgax-DTR BM chimeras. **(B–D)** Absolute number of distinct cDC subsets **(B)**, monocytes **(C)**, and neutrophils **(D)** in the dLN 24 h after i.m. immunization with OVA/gE/AS01 (0.5/2.5/2.5 µg) per injection site with or without prior DT-mediated depletion of CD11c^+^ cells. **(E)** Schematic representation of Itgax-DTR BM chimeras treated 12 h prior to or 24 h after OVA/gE/AS01 (0.5/2.5/2.5 µg) per injection site for both sensitization and challenge. **(F, G)** Percentage OVA_257–264_ MHCI-Tetramer^+^ CD8^+^ T cells in the blood **(F)** and spleen **(G)** of Itgax-DTR BM chimeric mice 21 days after i.m. immunization at d0 and challenge at d14 with OVA/gE/AS01 (0.5/2.5/2.5 µg) per injection site and DT treatment. **(H)** Percentage IFN-γ and/or IL2 producing CD8^+^ T cells upon OVA restimulation in the spleen of Itgax-DTR BM chimeric mice 21 days after i.m. immunization at d0 and challenge at d14 with OVA/gE/AS01 (0.5/2.5/2.5 µg) per injection site and DT treatment. **(I)** Percentage IFN-γ and/or IL2 producing CD4^+^ T cells upon VZV gE restimulation in the spleen of Itgax-DTR BM chimeric mice 21 days after i.m. immunization at d0 and challenge at d14 with OVA/gE/AS01 (0.5/2.5/2.5 µg) per injection site and DT treatment. **(J)** Total VZV gE specific IgG levels in the serum of Itgax-DTR BM chimeric mice 21 days after i.m. immunization at d0 and challenge at d14 with OVA/gE/AS01 (0.5/2.5/2.5 µg) per injection site and DT treatment. Data are representative of 4 independent experiments (n = 2–5 mice per group). Error bars indicate mean ± SEM **(B–J)**. Data analyzed with a One-way ANOVA and Sidak’s multiple comparison test *p < 0.05, **p < 0.01, ns, non-significant.

To examine the non-redundant role of CD11c^+^ cells, such as cDCs and MCs, in mounting the adaptive immune response, we measured different aspects of the immune response after Diphtheria toxin (DT)-mediated CD11c^+^ cell depletion. CD11c^+^ cell depletion was achieved by injecting DT into animals that had bone marrow transplants from mice transgenic for DTR driven by the *Itgax* promotor, driving expression of CD11c (**Figure 3A**) (27, 34, 35). DTR transgene expression rendered CD11c^+^ cells sensitive to the lethal effect of DT, while the bone marrow chimerism eliminated off-target DT toxicity in the colon of intact *Itgax*-DTR mice. In the absence of DT, immunization with AS01-adjuvanted vaccines in these chimeric mice led to an increase of all cDC subsets, monocytes, and neutrophils after 24 h, as expected (**Figures 3B–D**). The i.m. injection of DT 12 h before OVA/gE/AS01 immunization abrogated the accumulation of cDCs (**Figure 3B**) and monocytes (**Figure 3C**) in the dLN 24 h post-immunization, while neutrophils were less affected (**Figure 3D**). The reduced recruitment of monocytes after DT-mediated CD11c^+^ cell depletion could be related to their intermediate CD11c expression, or their dependence on CD11c^hi^ cells, such as cDCs, for recruitment.

Next, we investigated whether CD11c^+^ cells were necessary for the induction of antigen-specific T cells responses and antibodies. Chimeric mice were immunized twice at d0 and d14 with OVA/gE with or without AS01. DT was given either 12 h before or 24 h after each vaccine dose (**Figure 3E**). DT administration at -12 h led to the depletion of all three major cDC subsets in the dLN (**Figure 3B**). Mice depleted of CD11c^+^ cells before or after immunization with OVA/gE/AS01 failed to mount an antigen-specific CD8^+^ T cell response when compared to immunized mice not receiving DT treatment, as measured by the complete absence of OVA_257–264_-tetramer positive CD8^+^ T cells in the blood (**Figure 3F**) or in the spleen (**Figure 3G**), or IFN-γ/IL-2 production by OVA restimulated CD8^+^ splenocytes. (**Figure 3H**). We also measured cytokine responses upon stimulation with VZV gE peptide pool by CD4^+^ T cells in the spleen at day 21. Interferon-γ and IL-2 cytokine production by CD4^+^ T cells was reduced in mice depleted of CD11c^+^ cells, only when DT was administered before OVA/gE/AS01 administration, but not 24 h later, suggesting that priming of CD4^+^ T cells by CD11c^+^ cells occur mainly within 24 h after immunization (**Figure 3I**). Finally, the increase in anti-gE IgG titers in mice immunized with AS01-adjuvanted vaccine compared to non-adjuvanted group was reduced in DT-treated mice, although this was not reaching statistical significance (**Figure 3J**). Overall, these data point to a crucial role of CD11c^+^ cells in the induction of cellular immune responses driven by AS01.

### CCR2- and Flt3-Dependent Cells Are Required for Adaptive Immunity to AS01-Adjuvanted Vaccines

**Figure 4:**
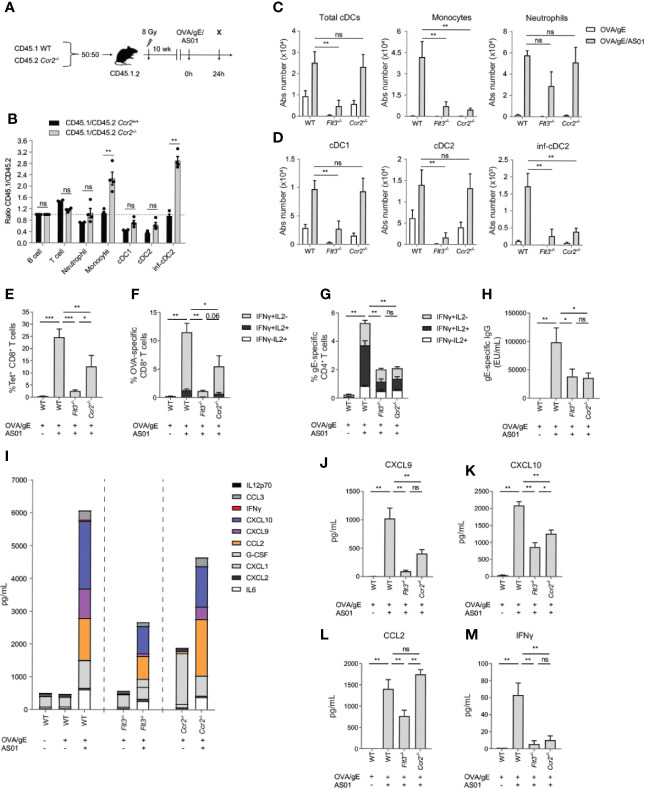
Both Flt3 and CCR2 dependent cells are required for optimal adaptive response driven by AS01. **(A)** Schematic representation of CD45.1 WT: CD45.2 Ccr2^-/-^ BM chimeras. **(B)** Normalized CD45.1/CD45.2 ratio relative to B cells of cell subsets in the dLN 24 h after i.m. immunization with OVA/gE/AS01 (0.5/2.5/2.5 µg) per injection site. **(C)** Absolute number of total cDCs, monocytes and neutrophils in the dLN of WT, Flt3^-/-^, and Ccr2^-/-^ mice 24 h after i.m. immunization with OVA/gE (0.5/2.5 µg) formulated in 2.5 µg AS01 or buffer per injection site. **(D)** Absolute number of different cDC subsets in the dLN of WT, Flt3^-/-^, and Ccr2^-/-^ mice 24 h after i.m. immunization with OVA/gE (0.5/2.5 µg) formulated in 2.5 µg AS01 or buffer per injection site. **(E)** Percentage OVA_257–264_ MHCI-Tetramer^+^ CD8^+^ T cells in the spleen of WT, Flt3^-/-^, and Ccr2^-/-^ mice 21 days after i.m. immunization with OVA/gE (0.5/2.5 µg) formulated in 2.5 µg AS01 or buffer per injection site. Same set-up as in **Figure 3E** without DT treatment. **(F)** Percentage IFN-γ and/or IL2 producing CD8^+^ T cells upon OVA restimulation in the spleen of WT, Flt3^-/-^, and Ccr2^-/-^ mice 21 days after i.m. immunization with OVA/gE (0.5/2.5 µg) formulated in 2.5 µg AS01 or buffer per injection site. Same set-up as in **Figure 3E** without DT treatment. **(G)** Percentage IFN-γ and/or IL2 producing CD4^+^ T cells upon VZV gE restimulation in the spleen of WT, Flt3^-/-^, and Ccr2^-/-^ mice 21 days after i.m. immunization with OVA/gE (0.5/2.5 µg) formulated in 2.5 µg AS01 or buffer per injection site. Same set-up as in **Figure 3E** without DT treatment. **(H)** Total VZV gE specific IgG levels in the serum of WT, Flt3^-/-^, and Ccr2^-/-^ mice 21 days after i.m. immunization with OVA/gE (0.5/2.5 µg) formulated in 2.5 µg AS01 or buffer per injection site. Same set-up as in **Figure 3E** without DT treatment. **(I–M)** Cytokine and chemokine levels in serum of WT, Flt3^-/-^, and Ccr2^-/-^ mice 24 h after i.m. immunization with OVA/gE (0.5/2.5 µg) formulated in 2.5 µg AS01 or buffer per injection site. Shown individually for CXCL9 **(J)**, CXCL10 **(K)**, CCL2 **(L)**, and IFN-γ **(M)**. **(A–M)** Data are representative of 4 independent experiments (n = 2–5 mice per group). Error bars indicate mean ± SEM. Data analyzed with a Two-way ANOVA **(B–D)** or One-way ANOVA **(E–M)** and Sidak’s multiple comparison test. *p < 0.05, **p < 0.01, ***p < 0.001, ns, non-significant.

The experiments in *Itgax*-DTR mice did not dissect which CD11c^+^ APC subsets was required for the adjuvant effect. MCs are typically defined by their dependency on the chemokine receptor CCR2, which is needed for egress of Ly6C^hi^ monocytes from the bone marrow. As a consequence, all of the monocyte progeny would be disadvantaged in *Ccr2^-/-^* mice, compared with WT mice, as seen in competitive bone marrow (BM) chimeric experiments (23, 26, 36). In addition, subtypes of tissue-cDC2s can also depend on CCR2 (37), especially during inflammation (23, 38). We therefore probed for the cell intrinsic role of CCR2 on cDC subsets in mice immunized with AS01-adjuvanted vaccines. In the dLN 24 h after OVA/gE/AS01 i.m. injection in WT : *Ccr2^-/-^* mixed BM chimeras (**Figure 4A**), cDC1s and cDC2s derived equally well from both donor BM compartments, whereas inf-cDC2s were generated more efficiently from the WT BM component (**Figure 4B**). As a positive control, monocytes were also mainly derived from the WT BM component. We therefore confirmed that monocytes and inf-cDC2s are the main subsets that are dependent on CCR2.

Flt3 is a hematopoietic cytokine receptor expressed by DC-progenitors, and *Flt3^-/-^* mice lack all subsets of cDCs as well as pDCs (18, 39). Comparisons of cDCs in *Flt3^-/-^* and *Ccr2^-/-^* mice can thus provide valuable insights into which of the cDC subsets is functionally required for AS01 adjuvant effect. As expected, *Flt3^-/-^* mice had a significant reduction of total cDC number in the dLN 24 h pi with AS01-adjuvanted vaccine (**Figure 4C**; left panel). In line with their pre-cDC origin this reduction was apparent for both cDC1 and cDC2 subsets in *Flt3^-/-^*, while these subsets were numerically conserved in *Ccr2^-/-^* mice (**Figure 4D**; left and middle panel). Strikingly, inf-cDC2s were reduced in both *Flt3^-/-^* and *Ccr2^-/-^* mice (**Figure 4D**; right panel). This is in line with previous work, demonstrating that inf-cDC2s are Flt3L dependent pre-cDC-derived cells that intrinsically depend on CCR2 (23). At the same time, CCR2-dependent monocytes were also reduced in immunized *Flt3^-/-^* mice, which could indicate that *Flt3*-dependent DCs are indirectly required for the recruitment of monocytes and monocyte-derived cells. Increase in neutrophil numbers in dLN after AS01 vaccination was not altered in both *Flt3^-/-^* and *Ccr2^-/-^* immunized mice (**Figure 4C**; right panel).

As specific tools to target inf-cDC2s are currently not available, we next compared the adaptive immune responses between WT mice, *Flt3^-/-^* mice in which all bona fide cDCs (and pDCs) will be targeted and *Ccr2^-/-^* mice in which mainly inf-cDCs, monocytes and MCs will be targeted. The induction of OVA_257–264_-tetramer positive CD8^+^ T cells was strongly inhibited in *Flt3^-/-^* mice and to a lesser extent in *Ccr2^-/-^* mice 7 days after a second dose of OVA/gE/AS01 (set-up as in **Figure 3E** without DT treatment) (**Figure 4E**). A similar pattern was observed when measuring cytokine production by CD8^+^ T cells upon OVA restimulation of splenocytes. (**Figure 4F**). Since CD8^+^ T cell responses were more strongly reduced in *Flt3^-/-^* compared to *Ccr2^-/-^* mice, it appears that the preserved cDC1s and cDC2s in *Ccr2^-/-^* mice can still induce some CD8^+^ T cell responses, whereas CCR2-dependent inf-cDC2s or MCs are still required to achieve the maximal response. In contrast, CD4^+^ T cell and antibody responses were equally reduced in both *Flt3^-/-^* and *Ccr2^-/-^* mice (**Figures 4G, H**). This indicates that an APC subset that relies on both Flt3/Flt3L and CCR2, such as inf-cDC2s, is contributing significantly to the activation and cytokine production by CD4^+^ T cells and antibody responses

Chemokines and IFN-pathway related cytokines are rapidly induced by AS01 at the injection site and dLN and early IFN response plays an important role in the effect on CD4^+^ T cell priming (27). Overall production of such pro-inflammatory cytokines was significantly reduced in the serum of *Flt3^-/-^* and *Ccr2^-/-^* mice, and in particular for IFN-dependent cytokines CXCL9 and CXCL10 (**Figures 4I–K**). Those cytokines are known to recruit lymphocytes *via* CXCR3 interaction and are associated with efficient induction of cellular immune responses (40). Furthermore, the ligand for CCR2, i.e. CCL2, was reduced in the serum of *Flt3^-/-^* immunized mice (**Figure 4L**), pointing to a role of DCs in the chemotaxis of CCR2-dependent cells. The prototypical Th1 cytokine IFN-γ was barely detectable in serum of both immunized *Flt3^-/-^* and *Ccr2^-/-^* mice (**Figure 4M**), whereas it was induced in AS01-immunized WT mice.

In conclusion, these results indicate that the adaptive immune responses induced by AS01-adjuvanted vaccines depend on cells that require both Flt3/Flt3L and CCR2.

## Discussion

In line with our previous work, we show here that the adjuvant system AS01 strongly impacts the innate immune response and leads to a substantial influx of activated cDCs 24 h pi to the dLN in mice (27). When tested separately, this immune enhancing effect was mainly mediated by MPL in AS01. In addition to inducing recruitment of cDC1s and cDC2s to the draining lymph node, AS01 also promoted recruitment of inf-cDC2s, a recently discovered pre-cDC derived cell type whose induction depends on type I IFN, Flt3L and CCR2 *in vivo* (23). Given the hybrid features of inf-cDC2s, such as the expression of Ly6C and activating Fc receptors CD64 and MAR-1, these cells have been misclassified as migratory MCs with APC capacity in earlier studies (25, 27, 41). However, true monocyte-derived MCs were barely detected within the migratory DC pool in the dLN of AS01 immunized mice after 24 h and therefore are unlikely to contribute significantly in T cell priming. In contrast, using an *ex vivo* antigen presentation assay 24 h after OVA/AS01 vaccination, we showed that cDC2s and inf-cDC2s are capable and equally efficient at priming both OVA-specific CD4^+^ and CD8^+^ T cells. Although we did not test this experimentally yet, we predict that inf-cDC2s could play an even more dominant role at time of a subsequent vaccination once antigen-specific antibodies are present and could promote the engulfment of opsonized antigen through Fc receptors expressed on inf-cDC2s. It is indeed known that antibodies can aid in the delivery of antigens to Fc-receptor bearing APCs in a process appreciated as the vaccinal effect (42) and that expression of high-affinity Fc receptors on inf-cDC2s direct antibody-complexed antigen in a very effective way to these cells (23).

Using an *in vivo Itgax*-DTR depletion model, we have demonstrated that CD11c^+^ cells play a non-redundant role in the elicited adaptive immune response driven by AS01. Depletion of CD11c^+^ cells prior to OVA/gE/AS01 immunization indeed abolished both CD4^+^ and CD8^+^ antigen-specific T cell responses, while depletion of DCs 24 h pi affected CD8^+^ but not CD4^+^ T cell responses. This is in line with previous observations showing that AS01 adjuvant effect is dramatically reduced, when the antigen is given 24 h after AS01 administration, and in particular CD4^+^ T cells (27). Demonstrating the relative and non-redundant contribution of the individual cDC subsets in the induction of adaptive responses remains nevertheless challenging. Transgenic mouse lines which allow for selective depletion of DC subsets exist for cDC1s (e.g. *Xcr1*-Cre x *Irf8^fl/fl^* or *Xcr1*-DTR), but are currently lacking for (inf-)cDC2s, given the overlap in genes between (inf-)cDC2s and MCs. In the future, the development of new transgenic mouse lines using dual cassettes targeting non-overlapping gene modules between distinct cell subsets can help overcome current limitations.

We also found that antibodies were only partially affected in the absence of DC subsets, despite of a reduction in T cells. Similarly, mice lacking IFN-γ or depleted in NK cells have a profound reduction in cytokine production by antigen-specific CD4^+^ T cell promoted by AS01 but the impact of antibodies was limited (9). Therefore, additional pathways, involving direct B cell activation, may be involved in antibody production, independent of DC subsets and polyfunctional T cells.

Conventional DCs typically depend on Flt3/Flt3L (19), while dependency on CCR2 is widely used to define monocyte descent (26). Consequently, several papers have used *Ccr2^-/-^* mice to show that monocyte-derived progeny contributes functionally to a given phenotype (43–48). However, it has been shown that certain cDC2 subtypes also intrinsically depend on CCR2 for migrating into tissue (37), especially during inflammation, and therefore CCR2-dependency of a response cannot be considered equal to monocyte-dependency (23, 25, 38). Whereas AS01 immunized *Ccr2^-/-^* mice had particularly reduced numbers of inf-cDC2s, all cDC subsets were equally reduced in the dLN of *Flt3^-/-^* mice. Consequently, *Flt3^-/-^* mice mounted very poor antigen-specific effector responses to the extent of what was observed in the CD11c^+^ cell depleted mice. Despite normal numbers of cDC1s and cDC2s, *Ccr2^-/-^* mice failed to raise normal levels of antigen-specific antibodies after boost, and induce proper CD4^+^ and CD8^+^ T cell responses. Since CCR2-dependent MCs poorly migrate to the dLN after AS01 injection and have inert APC functions in other systems when carefully separated from inf-cDC2s (23), the dependency of inf-cDC2s on CCR2 offers a more plausible explanation why *Ccr2^-/-^* mice have impaired Th1 cell-mediated responses after immunization with AS01-adjuvanted vaccine and increased susceptibility to viral infections (36, 43, 44). In an attempt to experimentally prove the discrepancy between inf-cDC2s and MCs, we tried to directly probe for the APC capacity of MCs but were not able to sort enough MCs from the dLN of AS01 immunized mice. We would like to state that we do not exclude any role of monocytes or MCs in AS01 adjuvanticity. Here, we conclude that MCs are unlikely to substitute the role of bona fide cDCs in priming naive lymphocytes after AS01 adjuvanted vaccination *in vivo* since MCs poorly migrate to the dLN and fail to prime naive T cells *ex vivo*. We cannot exclude that since they mostly reside in tissues, MCs could serve as an APC orchestrating the local reactivation or differentiation of previously primed lymphocytes migrating in inflamed tissue.

We have previously shown that type I IFN-dependent chemokines CXCL9 and CXCL10 are mainly produced by inf-cDC2s (23). In line with this, these chemokines were reduced in both *Flt3^-/-^* and *Ccr2^-/-^* AS01 immunized mice, and we propose this is mainly caused by the reduction of inf-cDC2s in these mice. The expression of these chemokines by inf-cDC2s may even further enhance Th1 responses (40, 49), explaining in part why intracellular IFN-γ/IL-2 production was defective in CD4^+^ and CD8^+^ T cells of AS01-immunized *Ccr2^-/-^* and *Flt3^-/-^* mice. Moreover, the prototypical Th1 cytokine IFN-γ, was also reduced in serum of *Flt3^-/-^* and *Ccr2^-/-^* mice compared to wild-type counterparts 24 h pi with AS01. Finally, the chemokine ligand CCL2 (or MCP-1), the best-known ligand for CCR2, was reduced in the serum of *Flt3^-/-^* immunized mice, pointing to a role of DCs in the chemotaxis of CCR2 dependent cells, such as inf-cDC2s and monocyte-derived cells. The reduced production of this chemokine could explain the reduction of monocyte accumulation in the dLN of DC-depleted *Itgax*-DTR mice and *Flt3^-/-^* mice. This is in line with our previous work indicating that DCs can be an important source of CCL2 for monocyte recruitment early in the induction of an adaptive immune response (50).

Intrinsic type I IFN signaling on cDCs is required for the development, activation, and maturation of inf-cDC2s *in vivo*. In addition, the induction of Fc receptors on BM cDC2s cultured in Flt3L and stimulated by lipopolysaccharide (LPS), a TLR4 agonist like MPL, was dependent on a type I IFN autocrine loop (23). Similar effects could be elicited using IFN-γ. Therefore future experiments should address whether inf-cDC2s, and the adaptive immune response in general, elicited by AS01 critically depends on type I or II IFN signaling, particularly since AS01 has been shown to elicit an early interferon signature, shown to play a role in the induction of polyfunctional CD4^+^ T cells in mice (9, 51). This might have implications for patients with impaired IFN responses such as elderly and diabetics (52, 53), which are highly vulnerable for viral infections such as COVID-19 (54, 55). Yet it seems that AS01 is able to overcome this impairment since the AS01-adjuvanted recombinant Zoster vaccine is able to induce a sustained cell-mediated responses in older adults (56).

Finally, a hybrid inflammatory DC subset, called DC3, was recently identified in humans with shared characteristics between type 2 DCs and monocytes (such as FcϵRI, CD14, CCR2 and CD64) (57–60). These DC3s also secreted high levels of Th1 cell-polarizing cytokines (e.g. IL-12) and T cell-attracting chemokines (e.g. CXCL9 and CXCL10) (58) and, unlike monocytes or monocyte-derived cells, efficiently activated naïve T cells into IFN-γ producing effector cells. In addition, DC3s excelled in the induction of tissue-resident memory T (TRM) cells (58), which are a hallmark of protective anti-viral immunity induced by vaccination (61). Future studies in humans will have to address whether new adjuvant systems like AS01 also work by eliciting these potent functions of hybrid DC3s. Altogether recently defined murine inf-cDC2s and human DC3s, in addition to cDC1s and cDC2s, seem to play a crucial role in tissue immunity which defines them as promising targets for vaccines.

## Data Availability Statement

The raw data supporting the conclusions of this article will be made available by the authors, without undue reservation. Please direct any requests to the corresponding author BNL.

## Ethics Statement

The animal study was reviewed and approved by Ethische Commissie Dierproeven—faculteit Geneeskunde en Gezondheidswetenschappen Universiteit Gent.

## Author Contributions

AMD, CC, AC, KF, CB, HH, and BNL were involved in the conception and design of the study. KF, AC, and CB acquired the data, and AMD, CC, KF, AC, CB, HH, and BNL analyzed and interpreted the results. All authors were involved in drafting the manuscript or revising it critically for important intellectual content. All authors had full access to the data and approved the manuscript before it was submitted by the corresponding author. All authors contributed to the article and approved the submitted version.

## Funding

This work was supported by GlaxoSmithKline Biologicals SA. BNL is supported by a European Research Council (ERC) advanced grant, a concerted research initiative grant (GOA) from Ghent University, and an Excellence of Science (EOS) research grant. CB and KF are supported by grants from FWO (CB 1138019N; KF 11L3113N).

## Conflict of Interest

CC and AC are employees of the GSK group of companies. AD was an employee of GSK at the time of the study and owns GSK stocks.

The remaining authors declare that the research was conducted in the absence of any commercial or financial relationships that could be construed as a potential conflict of interest.

## Footnotes

1 https://www.fda.gov/vaccines-blood-biologics/vaccines/shingrix

## Abbreviations

For more information on LSIDs please see Inclusion of Zoological Nomenclature section of the guidelines.
