# Supplementary material for: CCR2- and Flt3-Dependent Inflammatory Conventional Type 2 Dendritic Cells Are Necessary for the Induction of Adaptive Immunity by the Human Vaccine Adjuvant System AS01
Source: Front Immunol. 2021 Jan 14;11:606805. doi: 10.3389/fimmu.2020.606805 (PMC7841299; doi:10.3389/fimmu.2020.606805)
Supplement: Supplementary file 1 [file Table_1.docx]

Supplementary table: antibodies

| **Antibody-fluorochrome (clone)** | **source** |
| --- | --- |
| CD64-BV711 (X54-5/7.1) | BioLegend |
| CD64-AF647 (X54-5/7.1) | BD Biosciences |
| FceR1a-biotin (MAR-1) | Thermo Fisher Scientific |
| CD3e-PE-Cy5 (145-2C11) | Thermo Fisher Scientific |
| Ly6G-APC-Cy7 (1A8) | BioLegend |
| Ly6G-FITC (1A8) | BioLegend |
| Ly6G-PE (1A8) | BioLegend |
| Ly6C-FITC (AL-21) | BD Biosciences |
| Ly6C-PE (AL-21) | BD Biosciences |
| Ly6C-AF700 (AL-21) | BD Biosciences |
| Ly6C-AF647 (HK1.4) | BD Biosciences |
| CD11c-PE-Cy7 (N418) | Thermo Fisher Scientific |
| CD11c-Efluor450 (N418) | Thermo Fisher Scientific |
| MHCII-APC-Efluor780 (M5/114.15.2) | Thermo Fisher Scientific |
| MHCII-FITC (M5/114.15.2) | Thermo Fisher Scientific |
| MHCII-AF700 (M5/114.15.2) | Thermo Fisher Scientific |
| CD11b-BV605 (M1/70) | BD Biosciences |
| CD11b-V450 (M1/70) | BD Biosciences |
| CD24-PE (M1/69) | Thermo Fisher Scientific |
| XCR1-BV660 (ZET) | BioLegend |
| XCR1-PE (ZET) | BioLegend |
| CD172a-PerCP-Efluor710 (P84) | Thermo Fisher Scientific |
| CD172a-PE-Cy7 (P84) | BioLegend |
| CD172a-APC (P84) | BD Biosciences |
| CD26-FITC or -APC (H194-112) | BD Biosciences |
| CD26-APC (H194-112) | BioLegend |
| CCR2-BV421 (SA203G11) | BioLegend |
| CD45.1-BV605 (A20) | BioLegend |
| CD45.1-PE (A20) | BD Biosciences |
| CD45.2-AF700 (104) | Thermo Fisher Scientific |
| CD45.2-PerCP-Cy5.5 (104) | BD Biosciences |
| CD86-PE-Cy7 (PO3) | BioLegend |
| CD197(CCR7)-PE-Cy7 (4B12) | Thermo Fisher Scientific |
| CD40-PE (3/23) | BD Biosciences |
| CD4-APC (RM4-5) | Thermo Fisher Scientific |
| CD4-PE-Cy7 (GK1.5) | Thermo Fisher Scientific |
| CD8a-PerCP-Cy5-5 (53-6.7) | Thermo Fisher Scientific |
| CD19-PE-Cy5 (Bio1D3(1D3)) | Thermo Fisher Scientific |
| CD19-BV510 (6D5) | BioLegend |
| CD19-APC-Cy7 (6D5) | BioLegend |
| Vb5-APC (MR9-4) | BioLegend |
| Va2-BV605 (B20.1) | BD Biosciences |
| CD90.2-PE (53-2.1) | BD Biosciences |
| IFN-γ-PE-Cy7 (XMG1.2) | Thermo Fisher Scientific |
| IL-2-APC (JES6-5H4) | BD Biosciences |
